# The mitochondrial genome of the Carniolan honey bee, *Apis mellifera carnica* (Insecta: Hymenoptera: Apidae)

**DOI:** 10.1080/23802359.2019.1671250

**Published:** 2019-09-30

**Authors:** Leigh Boardman, Amin Eimanifar, Rebecca T. Kimball, Edward L. Braun, Stefan Fuchs, Bernd Grünewald, James D. Ellis

**Affiliations:** a Honey Bee Research and Extension Laboratory, Entomology and Nematology Department, University of Florida, Gainesville, FL, USA;; b Independent Senior Research Scientist, Industrial District, Easton, MD, USA;; c Department of Biology, University of Florida, Gainesville, FL, USA;; d Institut für Bienenkunde, Polytechnische Gesellschaft, Goethe-Universität Frankfurt am Main, Oberursel, Germany

**Keywords:** Mitogenome, next-generation sequencing, C-lineage honey bee, *Apis mellifera carnica*

## Abstract

Sequencing the mitochondrial genome of the Carniolan honey bee, *Apis mellifera carnica,* revealed 16,358 bp, consisting of 13 protein-coding genes, 22 tRNA genes, two rRNA genes, and a control region. Phylogenetic analysis supported a close relationship to another south-eastern European (C-lineage) honey bee, *A. m. ligustica*.

The Carniolan honey bee, *Apis mellifera carnica* Pollmann, is native to south-eastern Europe. This large, brown-grey honey bee is popular with beekeepers due to its gentle nature and ability to tolerate colder temperatures. Previous mitochondrial DNA studies on *A. m. carnica* have investigated the restriction endonuclease cleavage maps (Smith and Brown 1990) and the population structure in geographic locations, often using the CO1-CO2 intergenic region that includes tRNA-Leu (e.g. Susnik et al. 2004; Muñoz et al. 2009; Nedić et al. 2009). Despite this, the complete mitochondrial genome for this *A. mellifera* subspecies is unavailable. Here, we report the sequenced mitogenome of a morphometrically identified *A. m. carnica* worker honey bee obtained from the Ruttner Bee Collection at the Bee Research Institute in Oberursel, Germany (Voucher No. 1668, Dr. M. Meixner, 1990, Austria, 46°37 N, 14°19 E. GenBank accession number MN250878).

Honey bee genomic DNA extraction, quantification, genomic library preparation, and PE-150-bp next-generation sequencing (Illumina Hi-Seq 3000/4000, San Diego, CA) were performed following Eimanifar et al. (2017). Bioinformatics were performed following Boardman et al. (2019). Briefly, sequencing quality was assessed using FastQC (Andrews 2010) and trimmed with Trimmomatic (Bolger et al. 2014). The resulting reads were mapped to reference mitochondrial genomes using Geneious Prime 2019.0.4 (Kearse et al. 2012). The reference genome with the highest pairwise identity used for mapping was *A. m. ligustica* (L06178). The assembled mitogenome was annotated with mitos2 (Bernt et al. 2013), and phylogenetic comparisons were performed using Mesquite v3.5 (Maddison and Maddison 2018) and RAxML 8.2.10 GTRGAMMA model (1000 bootstrap replicates, -f a option, Stamatakis 2014) on CIPRES Science Gateway v. 3.3 (Miller et al. 2010). *P*-distances were generated with PAUP 4.0a (Swofford 2003).

The mitogenome is 16,358 bp long (43.2% A, 41.6% T, 9.6% C, and 5.5% G), and the location of genes and RNAs resembles arthropod mitogenomes. Of the 13 protein-coding genes (PCGs), nine are encoded on the light strand (*nad2*, *co1*, *co2*, *atp8*, *atp6*, *co3*, *nad3*, *nad6*, and *cytb*), and four (*nad1*, *nad4*, *nad4l*, and *nad5*) on the heavy strand. *Atp8* and *atp6* share 19 nucleotides. The start codons from PCGs varied – six genes start with ATT, four with ATG, two with ATA, and one with ATC – while all PCGs end with TAA stop codon. The 22 transfer RNAs (tRNAs) lengths varied from 63 bp (tRNA-Ser and tRNA-Gln) to 78 bp (tRNA-Thr). Both the 16S ribosomal RNA (rRNA) (1371 bp, 44.1% A, 40.5% T) and 12S rRNA (785 bp, 39.7% A, 41.9% T) genes are found on the heavy strand. The control region is 832 bp and AT-rich (50.6% A, 45.2% T).

Phylogenetic analysis comparing the *A. m. carnica* mitogenome to 20 other *Apis* honey bee mitogenomes available on GenBank (Figure 1) revealed that it clustered with the C- and O-lineage honey bees *A. m. ligustica* (*P*-distance = 0.0016) and *A. m. meda* (*P*-distance = 0.0024), respectively. This matches the relationship between these lineages found in previous work (e.g. Whitfield et al. 2006; Han et al. 2012; Wallberg et al. 2014). The other honey bees, mainly from Africa (A-lineage), have *P*-distances greater than 0.01. Sequencing additional mitogenomes from other European and Middle Eastern subspecies is essential to further unravelling mitogenome evolution and diversity in *Apis*.

**Figure 1.**
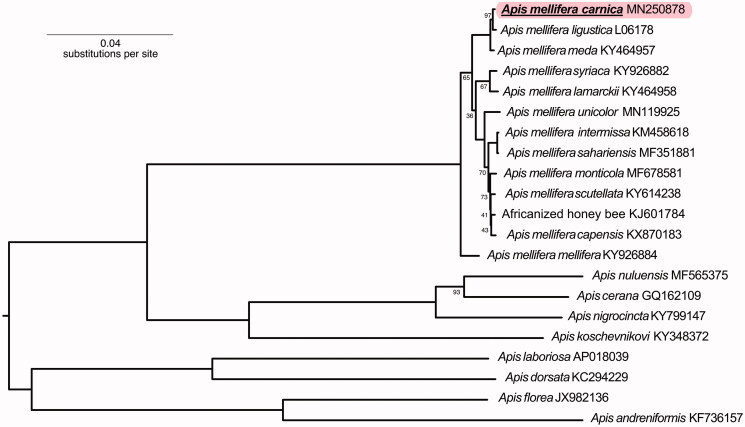
Phylogenetic tree showing the relationship between *A. m. carnica* (GenBank: MN250878) and 20 other *Apis* honey bees. Mitochondrial genomes consisting of 13 protein coding genes and 2 rRNAs were used to build the tree. The tree is midpoint rooted. The node labels indicate bootstrap values and unlabelled lineages are 100%.
